# Exosomes from antigen-pulsed dendritic cells induce stronger antigen-specific immune responses than microvesicles *in vivo*

**DOI:** 10.1038/s41598-017-16609-6

**Published:** 2017-12-06

**Authors:** Casper J. E. Wahlund, Gözde Güclüler, Stefanie Hiltbrunner, Rosanne E. Veerman, Tanja I. Näslund, Susanne Gabrielsson

**Affiliations:** Immunology and Allergy Unit, Department of Medicine Solna, Karolinska Institutet KS L2:04, SE-17176 Stockholm, Sweden

## Abstract

Extracellular vesicles (EV), including exosomes and microvesicles (MV), represent a rapidly expanding field of research with diagnostic and therapeutic applications. Although many aspects of EV function remain to be revealed and broad investigations are warranted, most published findings focus on only one vesicle category or a non-separated mix of EVs. In this paper, we investigated both MVs and exosomes from Ovalbumin (OVA)-pulsed dendritic cells for their immunostimulatory potential side-by-side *in vivo*. Only exosomes induced antigen-specific CD8^+^ T-cells, and were more efficient than MVs in eliciting antigen-specific IgG production. Further, mainly exosome-primed mouse splenocytes showed significant *ex vivo* interferon gamma production in response to antigen restimulation. Exosomes carried high levels of OVA, while OVA in MVs was barely detectable, which could explain the more potent antigen-specific response induced by exosomes. Moreover, exosomes induced increased germinal center B cell proportions, whereas MVs had no such effect. Immunisation with both vesicle types combined showed neither inhibitory nor synergistic effects. We conclude that DC-derived MVs and exosomes differ in their capacity to incorporate antigen and induce immune responses. The results are of importance for understanding the role of EVs *in vivo*, and for future design of vesicle-based immunotherapies and vaccines.

## Introduction

Extracellular vesicles (EV) have been studied as drug delivery vehicles, vaccine- and immune therapy tools and as carriers of biomarkers^1–3^. EVs are released from all cell types yet investigated but of varying amount and content depending on the state of the parent cell. The mechanisms driving EV formation, release and uptake are only partly understood. Silencing Rab GTPases involved in vesicle release^4^, or endosomal sorting complexes required for transport (ESCRT) components involved in vesicle formation^5^ has revealed redundancies in EV formation pathways. EVs are defined by differences in vesicle biogenesis, size and density^2,6^, and are generally assigned to either of three major categories; endosomally derived exosomes, cell surface-shed microvesicles (MV) (also referred to as “ectosomes” or “microparticles”)^7^, and apoptotic bodies. Exosomes are 30–150 nm in diameter, mainly pellet at 100´000 g and originate from multivesicular bodies (MVB) in endosomal pathways, whereas MVs are described as 0.1–1 μm in diameter, mainly pelleting at 10´000 g and formed by direct budding from the cell membrane. The idea that exosomes could be used in immune therapy arose when Raposo *et al*. showed that exosomes carry peptide/MHC complexes and prime specific immune responses^8^. Exosomes from dendritic cells (DC) were later shown to carry CD54, CD86 and MHC class I and II^9^ and to be able to induce antigen-specific tumour regression in a murine cancer model^10^. Further, autologous DC-derived Exo (DC-Exo) tested clinically in phase I cancer trials were well tolerated^11,12^. We have previously shown that strong Th1-skewed^13^ immune responses are induced by DC-Exo in mice, where the T cell response is B cell-dependent^13,14^. Furthermore, we have shown that T- and B cell responses to exosomes *in vivo* are mainly dependent on the presence of whole OVA antigen rather than MHC-peptide on the exosomes^15^. In addition, T- and B cell responses can be further potentiated with exosome-associated alpha-galactosylceramide^16^, a CD1d ligand activating NKT cells. Exosome-based immune therapy has also been applied to infectious disease vaccine models for Mycobacteria^17^, avian parasites^18^, Diphteria^19^ and *Leishmania major* ^20^. Similar to exosomes, MVs are present in body fluids and carry proteins, lipids and nucleic acids^2,21^. Although it is known that both MVs and exosomes can be released from the same cells^22^, MVs have not been as intensely investigated as exosomes, and side-by-side comparisons performed *in vivo* are rare. Kanada *et al*. showed, that MVs but not exosomes could transfer functional plasmid DNA *in vivo* ^23^, and other findings revealed overlapping but also distinct proteomic compositions of MVs and exosomes^24^. Taken together, it is highly likely that both vesicle categories have immunostimulatory functions *in vivo*. In the present paper we have, for the first time, investigated the *in vivo* immunostimulatory effects of exosomes and MVs derived from the same antigen-pulsed DCs, as a model for EV-based therapy. We found that the two vesicle types have clearly overlapping features, in terms of size distributions and immunostimulatory molecules. Despite the similarities, we found that exosomes and MVs differ greatly in capacity to incorporate exogenous antigen and to induce cellular and humoral immune responses *in vivo*.

## Results

### Exosomes and MVs from antigen-pulsed dendritic cells are equally enriched in immunostimulatory molecules

**Figure 1:**
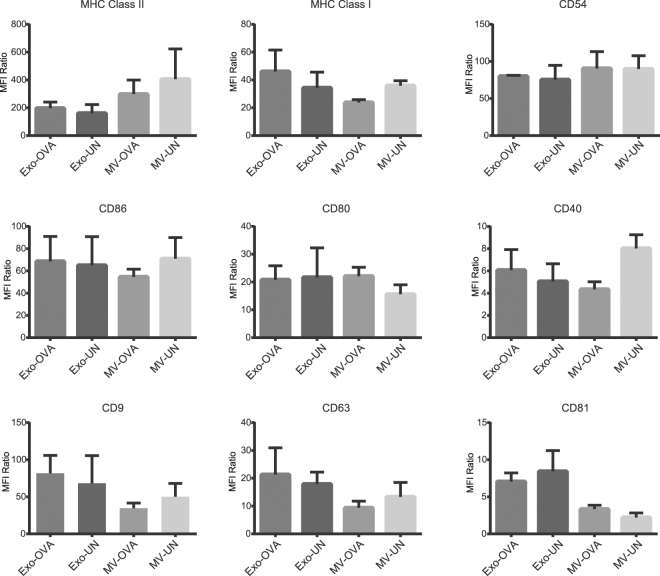
Exosomes and microvesicles from OVA-pulsed dendritic cells (DC) display similar levels of immunorelevant markers and tetraspanins. Using polystyrene-latex coated with anti-MHC class II antibodies, vesicles corresponding to the same total protein amounts were captured and analysed by flow cytometry. Quantified expression levels in mean fluorescence intensity of MHC class I and II, the adhesion molecule CD54, costimulatory CD86, CD80 and CD40 as well as the tetraspanins CD9, CD63 and CD81. All levels are MFI ratio of each marker normalised to isotype controls, conducted on four batches of exosomes (Exo) and microvesicles (MV) from OVA-pulsed (OVA) or un-pulsed DCs (UN).

Presence of MHC class II has been shown on both large and small DC-derived EVs^24^, anti-MHC class II beads were therefore used to capture EVs for flow cytometric characterisation. Exosomes and MVs displayed similar surface expressions of MHC class I, class II, costimulatory molecules CD40, CD80 and CD86, as well as of CD54 (Fig. 1), a glycoprotein with high affinity for lymphocyte function-associated antigen 1 (LFA-1)^25^. The expression of CD81 shows trends of lower levels in MVs, but is not significantly different (p = 0.062). Quantifications based on mean fluorescence intensity for four batches of EVs showed OVA-pulsing had no significant effect on the expressions of the markers analysed (Fig. 1 and histogram-based overview in Supplementary Fig. S1).

### Exosomes and MVs have overlapping size distributions and visual characteristics

**Figure 2:**
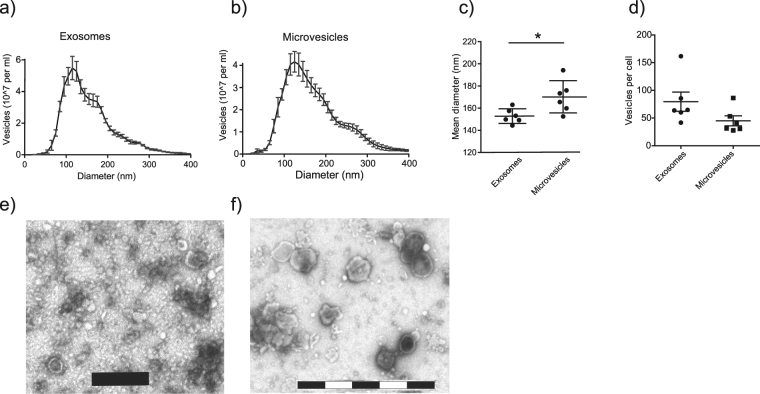
Microvesicles and exosomes have similar sizes and characteristics. Nanoparticle Tracking Analysis (NTA) showed overlapping size distributions of exosomes (**a**) and microvesicles (MV) (**b**), a mean diameter of 153 nm for exosomes and 170 nm for MVs (p = 0.026, **c**) and equal numbers of vesicles released per cell (**d**). Results are pooled of three independent experiments conducted on in total six separate preparations per vesicle type. Five 60 second recordings per sample were batch analysed. Transmission electron microscopy showed typical characteristics of exosomes (**e**) and similar, but generally larger sizes of MVs (**f**). Size bars (in **e** and **f**) are 200 nm per segment (black or white).

Nanoparticle tracking analysis (NTA) of the vesicles revealed similar size distributions of exosomes and MVs (Fig. 2A,B). Comparing mean diameters, MVs were slightly larger with a mean of 170 nm compared to 153 nm for exosomes (p = 0.026, Fig. 2C). When normalising the quantified vesicle numbers to the number of cells from which they were released, equal numbers of exosomes and MVs were noted per producing cell (Fig. 2D). Further, transmission electron microscopy (TEM) imaging of the vesicle preparations showed typical characteristics for exosomes (Fig. 2E), MVs displayed similar characteristics but generally larger sizes (Fig. 2F).

### Exosomes, but not MVs, induce splenic cell population changes including OVA-specific CD8^+^ T cells

**Figure 3:**
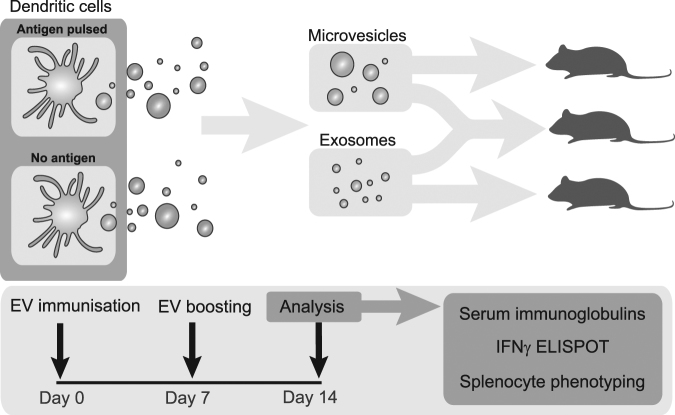
Experimental layout for *in vivo* analysis of immune responses to antigen-loaded extracellular vesicles. Exosomes and microvesicles from antigen-pulsed or non-pulsed bone marrow-derived dendritic cells were isolated, and injected at days zero and seven. On day 14, blood and spleens were analysed for serum immunoglobulins, spleen immune cell characterisation and interferon gamma (IFNγ) response to *ex vivo* antigenic restimulation.

**Figure 4:**
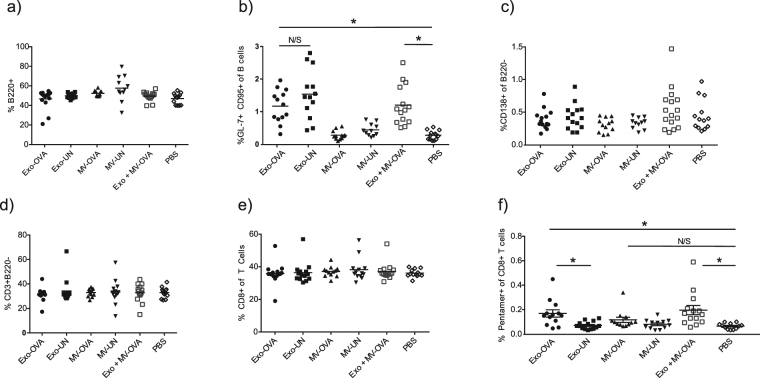
OVA-loaded exosomes (Exo-OVA) but not microvesicles (MV-OVA) induce immune cell population changes *in vivo*. Exo-OVA, MV-OVA or a combination thereof was injected on day zero and day seven in C57Bl/6 mice, and the splenocytes were characterised by flow cytometry on day 14. Percentages of B220^+^ B cells (**a**), GL-7^+^CD95^+^ germinal center B cells (**b**), CD138^+^ plasma cells (**c**), CD3^+^B220^-^ T cells (**d**), CD3^+^CD4^<sup>−</sup>^ CD8^+^T cells (**e**) and OVA-specific CD8^+^T cells (**f**) are displayed. Control groups were PBS, non-OVA loaded (UN) vesicles and PBS alone. Results are pooled from at least three experiments. (n = 4–6 per group per experiment). *Indicates p < 0.05, **indicates p < 0.01 and ***indicates p < 0.001, one-way ANOVA test with Dunn’s multiple correction.

To investigate the *in vivo* effects of the vesicles, exosomes and MVs isolated from OVA-pulsed BMDCs were injected intravenously in syngeneic C57Bl/6 mice. Immunisations were conducted on day zero and seven, with the immune response investigated on day 14 on splenic cells and serum immunoglobulins (Fig. 3). No significant differences were induced by exosome or MV immunisations regarding total numbers of B cells (Fig. 4A), but a significant increase in germinal center B cells (GCB) were found in all animals immunised with exosomes, regardless of antigen loading (p < 0.009 for all significant differences, Fig. 4B), suggesting that exosomes per se are potent GCB cell activators. Plasma cell levels did however not change in any groups (Fig. 4C). Proportions of T cells and CD8^+^ T cells did not change for any groups (Fig. 4D,E). However, increased levels of OVA pentamer positive CD8^+^ T cells were induced by exosomes (p = 0.0086) as well as the combined vesicle group (p = 0.0013) compared to PBS control, whereas MVs alone had no such effect (Fig. 4F).

### Exosomes induce higher levels of OVA-specific IgG antibodies compared to MVs

**Figure 5:**
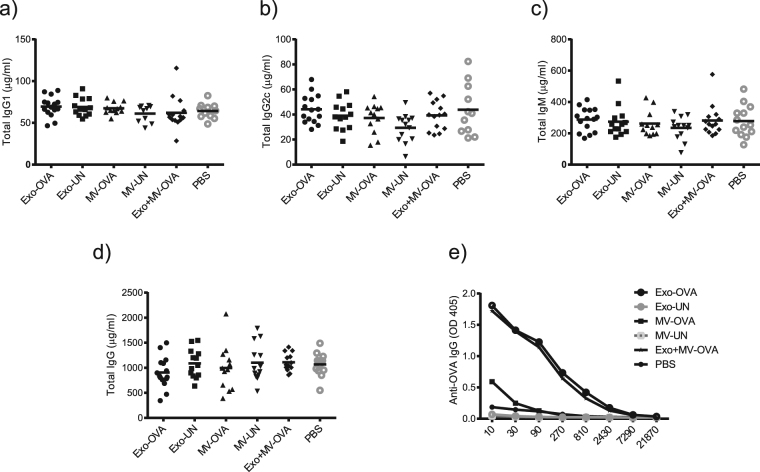
Exosomes induce higher antigen-specific IgG levels compared to microvesicles. Day 14 serum levels of mice immunised with exosomes (Exo-OVA), microvesicles (MV-OVA) or a mix of both vesicle types from 18 million OVA-pulsed dendritic cells was analysed for levels of total IgG1 (**a**), total IgG2c (**b**), total IgM (**c**), total IgG (**d**), and OVA-specific IgG (**e**). Results are pooled from three independent experiments (n = 4–7 per group per experiment). Control groups were non-antigen loaded (UN) vesicles and PBS. *Indicates p < 0.05, ***Indicates p < 0.001, one-way ANOVA test with Dunn’s multiple correction.

Serum immunoglobulin levels measured by ELISA on day 14 showed that total IgG1, IgG2c and IgM levels were not significantly different between any of the groups (Fig. 5A–C). Further, total IgG levels were not altered in any of the groups (Fig. 5D), however both MV and exosome-immunised mice showed higher amounts of OVA-specific IgG compared to control mice, with exosomes inducing the clearly strongest effect (Fig. 5E).

### Exosomes are superior to MVs in inducing T cell responsiveness to antigen restimulation

**Figure 6:**
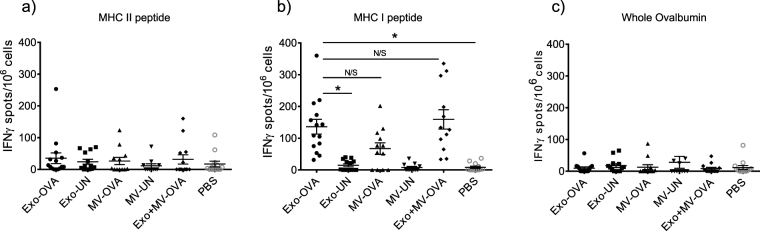
Splenocytes from all mice injected with OVA-loaded exosomes (Exo-OVA), and majority of those injected with microvesicles (MV-OVA) are responsive to antigenic restimulation. Splenocytes from mice immunised with either Exo-OVA, MV-OVA or a combination of the two were restimulated in an IFNγ ELISPOT. Numbers of IFNγ-producing units were counted after restimulation with the MHC class II-associated OVA_323–339_ peptide (**a**), the MHC class I-associated OVA peptide SIINFEKL (**b**), or with whole OVA protein (**c**). Control groups were immunised with vesicles without antigen (Exo-UN, MV-UN) or PBS. Results are pooled from at least three independent experiments (n = 4–7 per group per experiment). *Indicates p < 0.05 and ***Indicates p < 0.001, one-way ANOVA test with Dunn´s multiple correction was used.

To investigate lymphocyte responsiveness *ex vivo*, splenocytes from PBS-, exosome- or MV-injected mice were restimulated with either the immunodominant MHC class I-restricted peptide SIINFEKL, the immunodominant MHC class II-restricted OVA_323–339_ peptide or the full length Ovalbumin in an IFNγ ELISPOT. The MHC class II-restricted OVA peptide restimulation induced no IFNγ production above background levels (Fig. 6A), but in the presence of the SIINFEKL peptide, exosome-immunised mouse splenocytes responded with higher numbers of IFNγ-producing cells compared to PBS injected mice (p = 0.0002) (Fig. 6B). Splenocytes from MV-injected mice showed no overall significant differences to PBS control in a global statistic test, but 8 of 15 mice responded stronger than any control mice (PBS or unloaded vesicles) and there was no significant difference to Exo-immunised mice (Fig. 6B), indicating a more heterogenous response to the MVs. This was not batch-dependent, since both high- and low responder mice were seen in all experiments. Restimulation with the whole OVA antigen induced no detectable IFNγ production by splenocytes from any of the immunised mice (Fig. 6C).

### Endosomal proteins and antigen levels distinguish exosomes from MV

**Figure 7:**
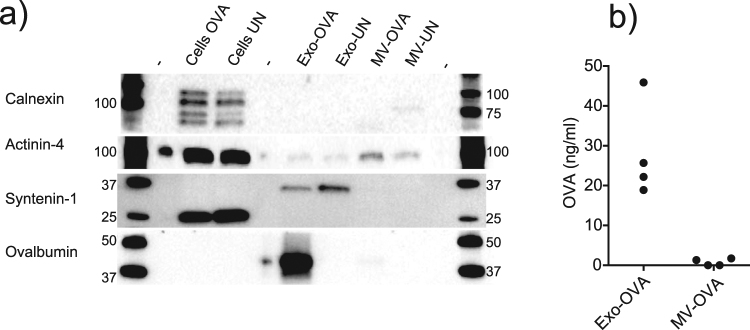
Exosomes are enriched in endosomal protein and incorporated antigen compared to microvesicles. (**a**) Western blot analysis of dendritic cell (DC) lysates and DC-derived vesicles for (from top) endoplasmic reticulum-associated Calnexin, MV-associated Actinin-4, ALIX-interacting Syntenin-1, Ovalbumin. (**b**) ELISA analysis of surface-oriented levels of Ovalbumin on four separate batches of exosomes and microvesicles. Representative of two experiments (**a**) or pooled result of four experiments (**b**). For b, a two-tailed t-test (Kruskal-Wallis) was performed. Uncropped gel images are displayed in Supplementary Figure S3.

To verify the different EV subtypes, and to understand the immune responses to the vesicles, a number of proteins in cell and vesicle lysates were analysed by Western blot. The endoplasmic reticulum-associated marker Calnexin was found only in cell lysates and not in EV preparations, indicating pure vesicle isolations (Fig. 7A, top). Distinguishing exosomes from MVs based on specific markers is challenging, we analysed the relationship of alpha-actinin 4 and syntenin-1, as suggested to be inversely expressed by small and large EV^24^. Actinin-4 was found in MVs but not exosomes and vice versa for Syntenin-1 (Fig. 7A), indicating clearly distinct vesicle populations. Further, since MVs were less potent in activating antigen-specific CD8^+^ T cells and OVA-specific IgG antibodies, we investigated the antigen content of both vesicle types. Whole OVA protein was found strongly enriched in exosomes, but could hardly be detected in MVs by Western blot (Fig. 7A). As EVs may interact differently with immune cells depending on surface antigen expression, we also investigated the amounts of OVA on the surface by ELISA. Again we found OVA protein on exosomes, whereas OVA levels in MVs were at the limit of detection (average levels 28,2 ng/ml for exosomes and 0,76 ng/ml for MVs, p = 0,029, Fig. 7B). Thus, OVA is mainly enriched in exosomes rather than in MVs.

## Materials and Methods

### Mice

For generation of bone marrow-derived dendritic cells (BMDC) and for immunisations, 7–9 week old female wild-type C57Bl/6 mice (Taconic, Denmark), were imported and kept under specific pathogen free conditions, and treated in accordance with rules and regulations as well as in line with the ethical permit approved by the Northern Stockholm ethical committee, Sweden.

### Generation and isolation of extracellular vesicles

BMDCs were generated and pulsed with Ovalbumin as previously described^14^, non OVA-pulsed DCs were used as control. Exosomes and microvesicles (MV) were isolated from the BMDC supernatants by differential centrifugations, 300 g for 10 min and 3000 g at 4 °C for 30 min, followed by 10´000 g for 40 min to pellet MVs. The supernatant was finally centrifuged for 90 minutes at 100´000 g to pellet the exosomes. Both pellets were washed in PBS, resuspended in PBS and stored at −80 °C.

### Transmission Electron Microscopy

The vesicles were mounted on carbon coated copper grids, contrasted with uranyl acetate and examined in a transmission electron microscope. Post imaging adjustments of images were performed using Photoshop (Adobe), only to increase contrast and to insert size bars where applicable.

### Nanoparticle tracking analysis

Nanoparticle tracking analysis (NTA) was conducted using a Nanosight LM10 (Nanosight/Malvern, Worcestershire, UK) to determine the vesicle size distributions. Each sample was diluted to 1–8 * 10^8^ particles/ml and analysed in five 60 second recordings using camera level 13, detection threshold 3, auto minimum expected particle size and auto jump distance in NTA version 3.0.

### Flow cytometric characterisation of vesicles

Exosomes or MVs were incubated with polystyrene latex beads coated with anti-MHC class II (I-A/I-E) antibodies at 2 μg total vesicle protein per μl beads (x * 10^5^ beads) and stained with fluorophore conjugated antibodies, acquired in a BD LSRFortessa flow cytometer, and surface markers were normalised to isotype controls in FlowJo software (FlowJo LLC).

### Quantification of vesicle-associated OVA

Amounts of surface-oriented OVA on the vesicles were measured by ELISA. Plates were coated overnight with vesicles corresponding to 10 μg/ml protein, blocked and incubated with mouse anti-OVA antibodies (Nordic Biosite, Stockholm, Sweden) and secondary HRP-conjugated anti-mouse antibodies (Southern Biotech, Birmingham, USA).

### Western blot

Total protein was isolated from the vesicles by vortexing the EV suspensions diluted 1:1 in RIPA buffer with protease inhibitors (Roche) for 30 seconds, sonicating 5 min 1:1 in in five cycles. Isolated protein was measured using a DC protein assay (BioRad) and 20 micrograms of protein from each vesicle suspension were boiled in Laemmli buffer (BioRad) at 95 °C for 5 min. The proteins were separated using SDS-PAGE, and detected using primary antibodies against OVA (Nordic BioSite, Taby, Sweden), calnexin (Santa cruz), Syntenin-1 and α-actinin-4 (Abcam), and secondary ECL sheep anti-mouse conjugated to HRP (GE Healthcare).

### Immunisations and splenocyte characterisation

Vesicles released from 18 million cells (generally equivalent to 50 μg total exosome or MV protein) resuspended in 100 μl PBS were injected intravenously per C57Bl/6 mouse. PBS was used as control. Seven days post immunisation, another injection was performed to boost the immune response. Combined vesicle groups contained both exosomes and MVs from 18 million cells. Two weeks after the initial immunisation, the mice were euthanised and blood and spleens were collected. Single-cell suspensions of splenocytes were analysed by flow cytometry and by *IFNγ* ELISPOT as described previously^14^.

### Serum ELISA

ELISA was used to assess serum concentrations of total IgM, IgG, IgG1 and IgG2c as well as OVA-specific IgG in serum diluted 0–2100x for OVA-specific IgG, 100–6400x serial dilution for the other isotypes as previously described^13^.

### Statistics

All *in vivo* results are based on at least three separate experiments, each with a new batch of vesicles, *in vitro* data are based on at least three experiments each unless otherwise stated in the figure legends. Kruskal Wallis test with Dunn’s correction was used for all three-group (or more)-comparisons, whereas the Mann-Whitney test was used for two-group comparisons. P < 0.05 was considered significant. All data generated or analysed during this study are included in this published article including supplementary information files.

## Discussion

How EV induce antigen-specific immune responses is still unclear, and comparisons of MV- and exosome-induced immune responses have been lacking. We investigated the immunostimulatory capacities of Ovalbumin-loaded dendritic cell derived exosomes (Exo-OVA) and microvesicles (MV-OVA) side-by-side *in vivo*, and found Exo-OVA to be superior in inducing germinal center B (GCB) cells, antigen-specific CD8^+^ T cells as well as OVA-specific IgG production and response to *ex vivo* antigenic restimulation with a CD8^+^-restricted peptide. We found no differences in expressions of immunostimulatory or adhesive molecules between MVs and exosomes to explain the different effects. As reviewed by Bachmann and Jennings^26^, the size, charge, and surface molecule organisation of antigens are central in vaccine engineering and will affect antigen uptake by immune cells, and transport in secondary lymphoid organs. Particle size affects how nanoparticulate vaccines interact with cells of the immune system, how they are taken up and transported in blood and lymph, as well as their duration and clearance (reviewed in ref.^27^). Furthermore, DCs have been shown *in vitro* to most efficiently take up nanoparticles smaller than 500 nm^28^, and OVA-associated synthetic nanoparticles tested *in vivo* elicit stronger immune responses if they are 40–50 nm in size as compared to 100, 500 and 1000 nm^29^. However, we found MVs to be only slightly larger in average (170 nm) than exosomes (153 nm) and of similar size distribution in this study. It is therefore not likely that vesicle size only can explain the observed different efficacies of exosomes and MVs in this study. However, it is still possible that only a fraction of the exosome population, e.g. the smallest vesicles, induce most of the immunogenic effects, but to evaluate that *in vivo* would require novel purification procedures. One other factor affecting uptake is the level of the phospholipid phosphatidylserine (PS), which has been found in higher levels in larger compared to smaller platelet-derived EVs^22^. All EVs are enriched in phospholipids including PS^30^, and PS plays a central role in MV formation, however we saw no significant difference in PS levels between exosome and MV preparations (Supplementary Fig. S2).

We argue instead that the most plausible explanation for the greater potency of exosomes in our setting is due to the higher antigen load in exosomes compared to in MVs. Consequently, this opens for the question how the two vesicle types are loaded with antigen, and if the only parameter observed differing between them is the antigen load, would MVs be more potent if equipped with higher antigen levels? One suggestion close at hand for how vesicles are loaded would be that OVA is merely attached to EV during vesicle production and isolation, and that this would favor association of OVA to exosomes rather than to MVs due to higher pelleting speeds for exosomes. This is however less likely, as our protocols include changing cell culture media after antigen pulsing to non-OVA-containing media two days before harvest and vesicle isolation. Instead, we suggest that the exogenous antigen is packed into exosomes during their biogenesis, as exosomes are formed in maturing endosomes where also antigen loading into MHC takes place. During MV formation, however, plasma membrane and cytosolic material is incorporated, so for exogenous antigen to be loaded in MVs it must first escape endosomal routes and enter the cytoplasm. This is possible in our setting based on primary DCs, which are adapted to facilitate cross-presentation of antigen which includes cytosolic escape of antigen for proteasomal degradation^31^. With high antigen dose it is possible that the proteasomal activity is insufficient for processing all antigen, leading to incorporation of low levels of antigen into MVs. Further, as different vesicles are released constitutively or upon specific stimulus of the host cell^30^, the optimal time point for harvesting high yields of antigen-loaded EVs may not be the same for all vesicle subtypes. Our protocols are optimised for exosome release and then applied to MVs, but an optimal approach may be to find the most efficient time point for each EV subtype. As MV-associated OVA was barely detectable, we were surprised to see MVs induce antigen-specific responses, although at modest levels. In the antigen restimulation, eight of 12 splenocyte suspensions produced IFNγ higher than that of any control group (unloaded vesicles as well as PBS). The antigen-specific IgG induction by MVs is also above that of all control groups, leading us to believe that optimisation of antigen loading and isolation procedure of several EV subtypes may open further vesicle-based therapy opportunities.

The three completed clinical cancer trials on based on dendritic cell derived exosomes^11,12,32^ have all been lacking signs of distinct disease regression. Although many other aspects for improved approaches can be considered including use of whole antigen instead of peptides^13^, combinations of EV subtypes may add significantly to treatment efficacy. In the current paper, we could not see any synergistic effects when combining exosomes and MVs in terms of antigen-specific antibody induction or elevated antigen-specific CD8^+^T cells. However, we argue that it is possible that several EV subtypes would amplify an antigen-specific response if an optimal setting for such a combinatorial approach can be established, including higher antigen loading ov MVs. In line with an idea of broad investigations, Tkach *et al*.^33^ recently investigated functional characteristics of DC-derived EVs isolated by differential centrifugations and found clearly overlapping features of several EV subtypes including similar size distributions and molecular expressions. They also found vesicles corresponding to our MVs and exosomes to enter, activate and to induce *in vitro* proliferation of allogeneic CD4^+^T cells equally efficient. This is interesting in light of our recent data showing that allogeneic exosomes also can induce antigen-specific immune responses^15^, something which can be further explored in clinical settings. To date, one previous *in vivo* analysis comparing MVs and exosomes has been made, however, these EVs vere derived from a tumor cell line^34^. Interestingly the study showed that although MVs had lower antigen levels than exosomes, they induced a more potent tumor protection compared to exosomes. The exosomal antigen load was approximately twice as high, but MV-associated antigen was still clearly detectable by Western blot. In our study, we could barely detect MV-associated antigen, and only found modest immune responses to MVs, it is thus plausible that the antigen levels in EVs must be above a critical threshold to favor a specific immune response. Our current findings contribute with the crucial aspect of *in vivo* evaluations of vesicles released from primary DCs, which may take up and process antigen differently compared to cell lines. More studies are needed to determine the optimal cell source of EVs in therapeutic settings, and whether our data reflects distinct roles for MVs and exosomes *in vivo* in a normal immune response.

We were suprised to see that exosomes induced a clear elevation of germinal center B (GCB) cell proportions, and that even non OVA-loaded exosomes induced the same effect. As GCB cells proliferate first after antigen encounter and uptake, followed by co-stimulation by helper T (Th) cells^35^, the effect must be associated to an antigen other than OVA. We argue that the most likely source of other exogenous antigen is the fetal bovine serum (FBS) used in the BMDC culture media. A general approach has been adopted to remove bovine EVs from the FBS by ultracentrifugation before use in EV experiments to reduce contaminating EVs, but many other non-murine proteins and molecules are still present and may be loaded into exosomes. We have seen repeatedly that *in vivo* applications of EVs isolated from FBS-containing cell cultures induce antibodies directed towards bovine serum albumin (own unpublished observations), clearly demonstrating an immunogenic effect of the bovine serum. This would not be an issue in a human clinical trial with a controlled culture medium, but it could be argued that some of the immunogenic effects achieved in our settings may be due to bystander activation by additional antigens. This should be kept in mind when moving to clinical studies, where this could be exploited to further increase EV efficacy.

In conclusion, we conducted the first *in vivo* investigation of immunostimulatory effects of DC-derived MVs and exosomes side-by-side, and found significant differences. We show that exosomes induce higher levels of antigen-specific IgG, and only exosomes induce OVA-specific CD8^+^ T cell populations, likely due to larger amounts of antigen carried by exosomes. The results from us and others showing immunostimulatory effects of not only exosomes but also MVs strongly suggest that all EV types should be considered and evaluated for immune therapeutic purposes.

## Electronic supplementary material

Supplementary information
