# Supplementary material for: Exosomes from antigen-pulsed dendritic cells induce stronger antigen-specific immune responses than microvesicles in vivo
Source: Sci Rep. 2017 Dec 6;7:17095. doi: 10.1038/s41598-017-16609-6 (PMC5719080; doi:10.1038/s41598-017-16609-6)
Supplement: Supplementary file 1 — Supplementary information [file 41598_2017_16609_MOESM1_ESM.pdf]

Supplementary Information

Exosomes from antigen-pulsed dendritic cells induce stronger antigen-specific immune responses than microvesicles *in vivo*

Casper J.E. Wahlund, Gözde Güclüler, Stefanie Hiltbrunner, Rosanne E. Veerman, Tanja I. Näslund, Susanne Gabrielsson*

Supplementary Figure S1

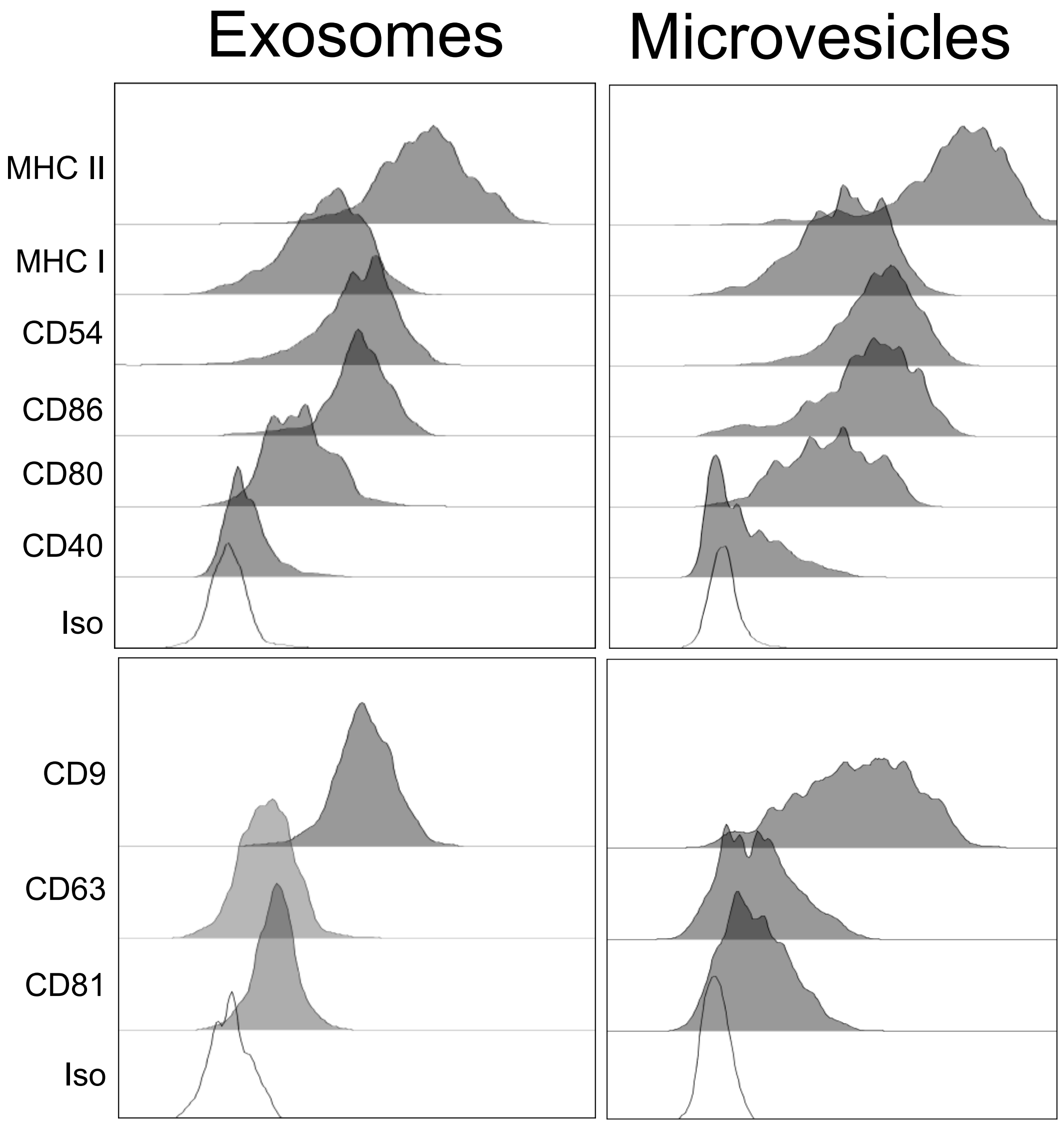

Supplementary figure S1

Exosomes or microvesicles from OVA-pulsed bone marrow-derived dendritic cells were bound to anti-MHC class II beads, stained for surface markers and aquired in a flow cytometer.

Supplementary figure S2

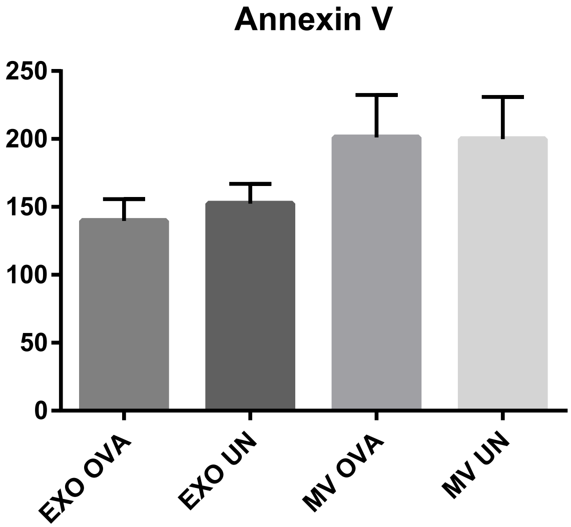

Supplementary Figure S2
Surface-oriented amounts of phosphatidylserine on exosomes (Exo) and microvesicles (MV) from OVA-pulsed (or UN-pulsed) bone marrow-derived dendritic cells. Vesicles were bound to anti-MHC class II coated beads and stained for Annexin V binding and aquired by a flow cytometer.

Supplementary figure S3

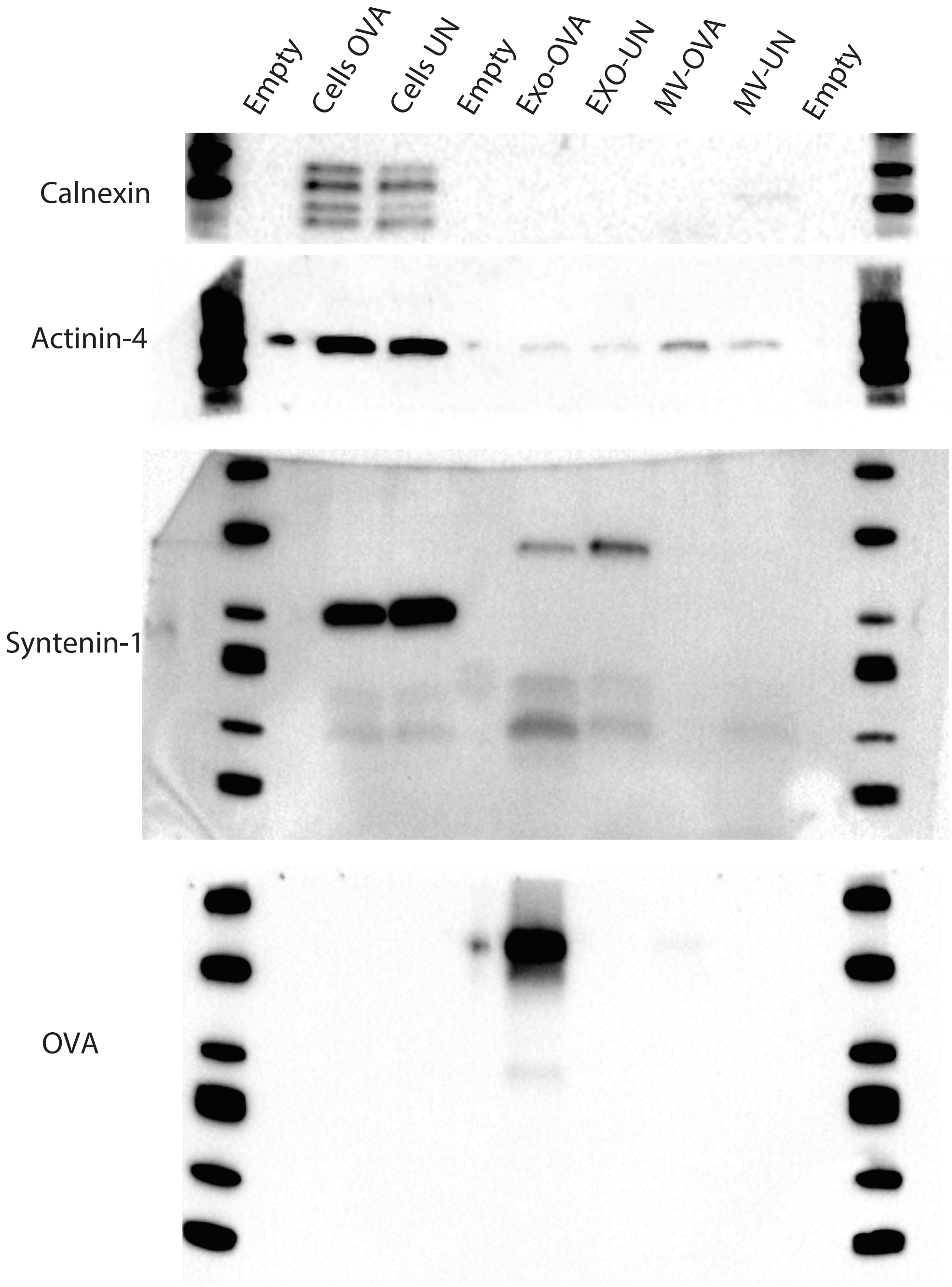

Supplementary Figure S3
Expanded non-cropped gel images from the Western Blot depicted in article figure 7a.
